# Massive induction of innate immune response to *Candida albicans* in the kidney in a murine intravenous challenge model

**DOI:** 10.1111/j.1567-1364.2009.00576.x

**Published:** 2009-11

**Authors:** Donna M MacCallum

**Affiliations:** Aberdeen Fungal Group, School of Medical Sciences, Institute of Medical Sciences, University of AberdeenForesterhill, Aberdeen, UK

**Keywords:** *Candida albicans*, experimental model, systemic infection, kidney, transcript profiling, innate immune response

## Abstract

In the experimental *Candida albicans* intravenous challenge model, the kidney is one of the main organs involved in disease. In progressive infection, fungal burdens are found to increase over time, with rapid increases occurring from 24 h postinfection. Renal transcriptional responses were analyzed at this time in the kidneys of mice infected by either a virulent or an attenuated *C. albicans* strain, allowing comparison of host responses in progressive and nonprogressive infection. The results of this study demonstrate that both infections share a common transcriptional response, consisting of functions associated with the acute-phase reaction. In addition, challenge with the virulent strain led to a massively increased expression of cytokine genes, other innate response genes and genes suggestive of initiation of the adaptive immune response. This immune response to *C. albicans* infection, which occurs only in progressive infection, may contribute to development of sepsis and, ultimately, host death.

## Introduction

Systemic fungal infections remain a significant cause of morbidity and mortality, with *Candida albicans* being the most common causative agent (Odds *et al.*, 2007; Bougnoux *et al.*, 2008; Caggiano *et al.*, 2008; Playford *et al.*, 2008;).

Intravenous *C. albicans* challenge in mice is a well-characterized model of severe clinical disseminated infection (MacCallum & Odds, 2005; Spellberg *et al.*, 2005;), used to investigate fungal virulence factors (Navarro-Garcia *et al.*, 2001; Brand *et al.*, 2004;) and host immune responses (Ashman, 2008; Romani, 2008; MacCallum *et al.*, 2009;). In this model, within minutes of entering the bloodstream, *C. albicans* is detectable in all major organs (MacCallum & Odds, 2005), with the kidneys and brain being the main target organs (Louria *et al.*, 1963; MacCallum & Odds, 2005;), reflecting the situation in the human host (Parker *et al.*, 1976; Odds, 1988;). In infection initiated by the virulent strain SC5314, renal fungal burdens increase from the time of infection, with rapid increases occurring from 24 h postinfection. Although the kidneys are the most heavily colonized organs, and some loss of renal function has been shown (Leunk & Moon, 1979; Spellberg *et al.*, 2005;), renal failure is not the main cause of death. Host deterioration, and eventually death, is due to a progressive sepsis, reflecting the clinical situation (Spellberg *et al.*, 2005).

Because of similarities to severe human disseminated infection, the mouse intravenous challenge model is ideal for studying the transcriptional response of the kidney to infection by *C. albicans*. DNA microarrays have been used previously to investigate the transcriptional responses of host cells (Prigneau *et al.*, 2003; Ishibashi *et al.*, 2004; Mullick *et al.*, 2004a; Barker *et al.*, 2005, 2008; Kim *et al.*, 2005b; Fradin *et al.*, 2006; Muller *et al.*, 2007) and fungal cells (Fradin *et al.*, 2003, 2005; Lorenz *et al.*, 2004; Sandovsky-Losica *et al.*, 2006; Sohn *et al.*, 2006; Fernandez-Arenas *et al.*, 2007; Thewes *et al.*, 2007; Zakikhany *et al.*, 2007; Walker *et al.*, 2009) during their interaction. Research has focused mainly on examining the responses of single host cell types, such as macrophages (Prigneau *et al.*, 2003; Lorenz *et al.*, 2004; Barker *et al.*, 2005; Kim *et al.*, 2005b;), neutrophils (Fradin *et al.*, 2005, 2006) and endothelial cells (Muller *et al.*, 2007; Barker *et al.*, 2008;). The aim of this study was to investigate the transcriptional responses of the whole kidney to infection by *C. albicans*, and to examine how these responses may relate to disease development, including progression of sepsis.

## Materials and methods

### *Candida albicans* strains and growth conditions

*Candida albicans* strains NGY152 (wild-type CAI4+CIp10, Brand *et al.*, 2004) and NGY355 (*pmr1*Δ null mutant, Bates *et al.*, 2005) were maintained routinely on Sabouraud agar at 4 °C, and stored long term in glycerol stocks at −80 °C. For the mouse intravenous challenge, inocula were prepared as described previously (Bates *et al.*, 2005).

### Experimental infection model

Female BALB/c mice (Harlan, UK) (average weight 20 g) were infected intravenously with an inoculum of 4.6 × 10^4^ CFU g^−1^ body weight. This inoculum level leads to a survival time of approximately 5 days for mice infected with the virulent strain NGY152 (MacCallum & Odds, 2005), but all animals infected with the attenuated strain NGY355 survived to the end of the 28-day experiment (Bates *et al.*, 2005). Groups of mice (*N*=3) were infected with *C. albicans* NGY152 (virulent), NGY355 (attenuated) or saline (controls). Actual inocula levels were determined by viable counts.

Mice were humanely terminated at 24 h. Kidneys were dissected and processed to provide portions for RNA extraction (flash-frozen in liquid nitrogen), histological analysis (formalin-fixed) and burden determination (homogenized in saline). Other organs (lung, liver, spleen and brain) were sampled and processed for organ burden determination as described previously (MacCallum & Odds, 2005). All work involving experimental animals was performed under UK Home Office licenses and regulations.

### RNA extraction

Frozen kidney pieces (approximately 50 mg) were ground to a fine powder in a Mikro-dismembrator U homogenizer (Sartorius Ltd, UK). The powder was resuspended in 1 mL TRIzol (Invitrogen, UK) and RNA extracted as per the manufacturer's instructions. RNA samples were DNase treated and purified using RNeasy mini cleanup columns (Qiagen, UK), the concentration was determined by spectrophotometry (Nanodrop ND-1000; Labtech International, UK) and the quality of the RNA was analyzed by a Bioanalyzer (Agilent Technologies, UK).

### Microarrays

For microarray analysis, pools of RNA for each condition were produced. Briefly, equal quantities (8 μg) of the three independent samples were mixed and made up to 24 μL. Each pool was then divided into three aliquots. For each treatment, two aliquots were used in the production of biotin-labeled cRNA. Standard Affymetrix protocols and kits (one-cycle cDNA synthesis, first strand synthesis, second strand synthesis, double-stranded DNA clean-up, GeneChip IVT synthesis and clean-up of biotin-labeled cRNA) were used to produce the labeled cRNA samples (http://www.affymetrix.com). From each cRNA sample, 20 μg was fragmented (1 × fragmentation buffer; 94 °C for 35 min), and 15 μg of this was used to produce a hybridization cocktail. Control noneukaryotic biotinylated and fragmented cRNAs (*bioB, C* and *D* from *Escherichia coli* and *cre* from bacteriophage P1) were added to the hybridization cocktail.

Cocktails for all six samples were hybridized to Affymetrix mouse genome 430A 2.0 arrays according to the manufacturer's instructions in an Affymetrix GeneChip hybridization oven 640. Arrays were washed in an Affymetrix GeneChip fluidics station and scanned with an Affymetrix GeneChip scanner 3000.

### Microarray gene expression data analysis

For each Affymetrix array, the CEL file was loaded into Genespring (Agilent Technologies). Data were normalized on a Per Chip basis (normalized to the 50th percentile) and on a Per Gene basis (normalized to the mean). Finally, data for *Candida*-infected kidneys were normalized on a Per Gene basis to control (saline-infected) samples.

Normalized data were analyzed by significance analysis of microarrays (http://www-stat.stanford.edu) to produce lists of genes with statistically significantly different expression levels, based on a false discovery rate value of 0. Gene lists were further trimmed to include only genes whose expression level differed at least 1.5-fold compared with uninfected controls. Gene trees were constructed in Genespring, with commonly regulated genes grouped together. Regulated genes were further divided into three large clusters with the three-cluster K-means function of Genespring.

Microarray data files [raw (CEL) and normalized files] were deposited in ArrayExpress (http://www.ebi.ac.uk/microarray-as/aer), accession number: E-MEXP-1458.

### cDNA synthesis and quantitative reverse transcriptase (QRT)-PCR

qRT-PCR with the Universal Probe Library system (Roche, UK) was used to verify differences in gene expression observed in microarray analysis.

Superscript II (Invitrogen) was used to synthesize cDNAs from 3 μg of each of the three independent RNA samples extracted from infected or control kidneys as per the manufacturer's instructions.

The oligonucleotide primers (Invitrogen) and Universal probes (Roche) used for qRT-PCR are listed in Table 1. PCR reactions (20 μL) contained 3 μL cDNA, 0.2 μL Universal probe, 0.2 μM forward primer, 0.2 μM reverse primer, 1 × probes Mastermix (Roche) and 1 × murine GAPDH endogenous control (PE Applied Biosystems, UK). Reactions were run on a LightCycler 480 (Roche); initial activation was carried out at 95 °C for 5 min, 50 amplification cycles of 95 °C for 10 s and 60 °C for 30 s and then a final cooling step at 40 °C for 10 s. For each gene, the copy number was determined from a standard curve and normalized to the GAPDH control. qRT-PCR reactions were carried out in triplicate for each of the three independent cDNA samples from each treatment group. Expression levels were compared using nonparametric statistical tests (Kruskal–Wallis and Mann–Whitney *U*-tests) due to unequal variances for the different groups.

**Table 1:** qRT-PCR oligonucleotide primers and Universal probes

Primer name	Sequence (5′–3′)	Universal probe
Tlr2F	TGCCCAGATGGCTAGTGG	51
Tlr2R	CAGAAACTATGATTGCGGACAC	
Tlr4F	GGACTCTGATCATGGCACTG	51
Tlr4R	CTGATCCATGCATTGGTAGGT	
Tlr13F	CTATGTGCTAGGAGCTTCTGAGAG	6
Tlr13R	TTCATCCTTCAAGCATCAGTGTA	
Il6F	GCTACCAAACTGGATATAATCAGGA	6
Il6R	CCAGGTAGCTATGGTACTCCAGAA	
Myd88F	TGACTTCCAGACCAAGTTTGC	80
Myd88R	GAATCAGTCGCTTCTGTTGGA	
Il1bF	TGTAATGAAAGACGGCACACC	78
Il1bR	TCTTCTTTGGGTATTGCTTGG	
Clec4nF	CAGTGAAGGGACTATGGTGTCA	78
Clec4nR	GCTCCAGAAGTTCTCCTTGGT	
Cxcl12F	TCCTCTTGCTGTCCAGCTCT	81
Cxcl12R	CAGGCTGACTGGTTTACCG	

### Bioinformatics: biological function and pathway analysis

Biological functions and pathways associated with the gene lists obtained from expression analysis were identified by means of Ingenuity Pathway Analysis (Ingenuity Systems; http://www.ingenuity.com) and the Database for Annotation, Visualization and Integrated Discovery (DAVID) (http://david.abcc.ncifcrf.gov) (Dennis *et al.*, 2003) bioinformatic resources. Both resources determine association by *P*-values, found by comparing genes on a list with the total number of genes for a pathway, and use knowledge databases that are continually updated from a number of sources.

### Histology

Sections (5 μm) were cut from paraffin wax blocks of formalin-fixed kidney portions. Sections were stained by periodic acid-Schiff to show the fungal cells clearly and poststained with hematoxylin to illustrate kidney morphology and immune infiltrates.

## Results

### Reduced virulence of strain NGY355 is evident at 24 h postinfection

In the murine model of systemic candidiasis, *C. albicans* strain NGY152 is virulent, attaining high renal fungal burdens and rapidly causing fatal infection (Brand *et al.*, 2004; MacCallum & Odds, 2005;). The second *C. albicans* strain, NGY355 (*pmr1*Δ), a mutant that lacks *N-* and *O-*linked mannan side chains in the cell wall, has been shown to be severely attenuated in virulence, both in terms of mouse survival times and of fungal organ burdens (Bates *et al.*, 2005; MacCallum *et al.*, 2009;).

Mice infected with either NGY152 (virulent) or NGY355 (attenuated) were sampled 24 h postinfection, when a rapid increase in kidney fungal burden is known to occur in kidneys infected by the virulent strain (MacCallum & Odds, 2005). In contrast, little change in kidney fungal burden was found at this time for the attenuated (*pmr1*Δ) strain (MacCallum *et al.*, 2009).

**Fig. 1:**
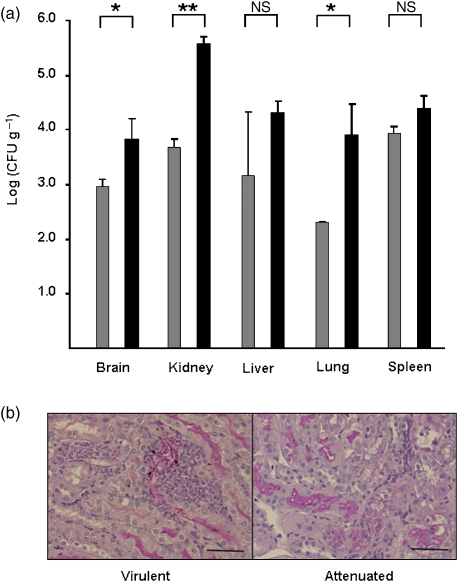
Disease pathology at 24 h postinfection is more severe in virulent strain infected mice. Groups of mice (*N*=3) were infected with either *Candida albicans* NGY152 (virulent), *C. albicans* NGY355 (attenuated) or saline as control, and sampled at 24 h postinfection. Disease progression was measured in terms of organ burdens and histopathology. (a) Higher fungal burdens were measured in the brain, lung and kidneys in mice infected with the virulent strain. Results represent the mean ± SD. Attenuated infection is represented by gray bars, virulent infection by black bars. Means were compared by Students *t*-test: ^*^P<0.05, ^**^P<0.01 and NS, significant. (b) Renal histopathology. Sections (5 μm) were stained with periodic acid-Schiff stain (fungal cells stained pink) and poststained with hematoxylin (shows tissue structure and stain immune cells blue/purple). Fungal cells (indicated by arrowheads) are obvious only in the kidneys of mice infected by the virulent strain. Scale bar represents 100 μm.

In this study, virulence differences between the two strains were evident at this early time, in terms of significantly higher burdens in the kidney, brain and lung (Fig. 1a) in mice infected with the virulent strain, with the greatest difference (approximately 100-fold) observed for the kidney.

The kidneys of virulent strain-infected mice contained filamentous fungal cells associated with leukocyte infiltrates (Fig. 1b), but there was little evidence of similar fungal lesions or immune cell infiltration in the kidneys of mice infected with the attenuated fungal strain. Control kidneys from saline-infected mice showed no evidence of fungal cells or immune cell infiltrates (data not shown).

### Renal gene expression changes associated with *C. albicans* infection

Because infection by the different *C. albicans* strains led to different renal phenotypes, i.e. immune infiltrate and lesion development, global transcript profiling of kidneys 24 h postinfection was used to reveal the local host responses underlying these different responses. Whole genome DNA microarrays were used to compare expression profiles of kidneys infected with either the virulent or the attenuated *C. albicans* strain relative to control, uninfected kidneys.

Results demonstrated that 2099 (5.4%) murine genes were upregulated in the kidneys of mice infected with NGY152 (virulent strain), compared with only 557 (1.4%) genes in the kidneys of mice infected with NGY355 (attenuated strain). The numbers of downregulated genes were similar to those upregulated, with 2069 (5.3%) for the virulent strain infection and 393 (1.0%) for the attenuated strain. There were 273 common upregulated and 286 common downregulated genes, representing the majority of those with altered expression levels in NGY355 infection. These commonly regulated genes are suggested to represent a core transcriptional response of the kidney to *C. albicans* infection.

**Fig. 2:**
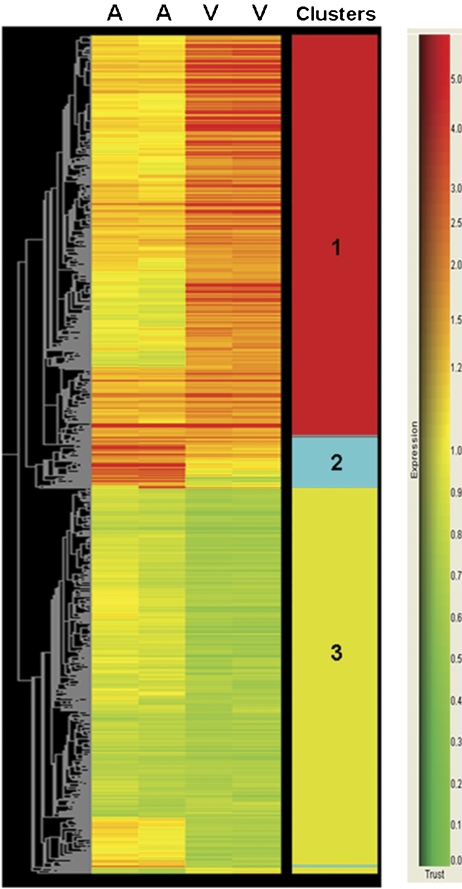
Genes with altered renal expression in systemic candidiasis form three distinct clusters. Differentially regulated genes in the kidneys of infected mice were analyzed by the Gene Tree function of Genespring to group genes regulated in a similar manner. Genes are colored by their fold regulation relative to control, as shown by the key on the right. K-means clustering separated the genes into three large clusters as numbered in the clusters column. Each column contains data from single microarray hybridizations, with V representing infection by the virulent strain and A representing infection by the attenuated strain.

All infection-regulated genes were grouped into thee large sets (Fig. 2) using clustering analysis based on K-means. Regulated genes common to both infections, and representative of the core response, were found in cluster 2, while genes more upregulated in the virulent strain infection formed cluster 1. Cluster 3 represented genes that were commonly downregulated or downregulated specifically in virulent strain infection.

### Biological functions and pathways associated with *C. albicans* infection

To identify biological functions and/or pathways associated with genes with altered expression in infected kidneys, results were analyzed by Ingenuity Pathway Analysis (http://www.ingenuity.com) and the DAVID (http://david.abcc.ncifcrf.gov) resource.

**Fig. 3:**
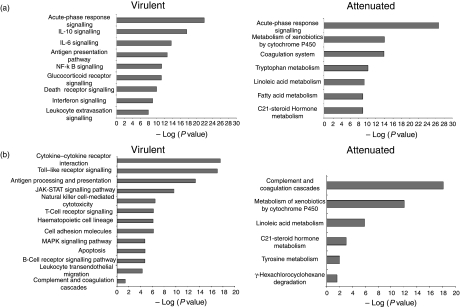
Infection by virulent or attenuated *Candida albicans* strains induces different responses in the kidney. Gene lists were analyzed by Ingenuity Pathway Analysis (included fold regulation) (a) or via the DAVID website (b). The most significantly regulated biological pathways or functions were determined by *P*-value.

Results demonstrated that both the attenuated and the virulent strains induced changes in gene expression associated with the acute-phase response (a generalized response to disturbances in physiological homeostasis) and complement and coagulation cascades (Fig. 3a and b). These responses represent the core response of the kidney to *C. albicans* infection, with their expression regulated in response to infection with either strain (cluster 2, Fig. 2).

In addition to the core response, infection with the attenuated strain induced regulation of genes in some metabolic pathways, whereas infection with the virulent *C. albicans* strain was strongly associated with regulation of genes involved in induced immune responses (Fig. 3a and b). Functions most strongly associated with virulent strain infection included cytokine–cytokine receptor interactions and a number of immune signaling pathways, such as interleukin (IL)-6, IL-10 and nuclear factor κB. In addition, gene expression changes linked to leukocyte movement were also associated with infection by the virulent strain. There was also evidence for initiation of an adaptive immune response with gene expression changes associated with antigen processing and presentation, and also signaling from both T-cell and B-cell receptors in the kidneys of mice infected with the virulent strain (Fig. 3b). Genes associated with the above functions were found in cluster 1 (Fig. 2), with little change in the expression of these same genes in kidneys infected by the attenuated strain.

Genes downregulated during infection, found in cluster 3 (Fig. 2), were associated with pyruvate metabolism, fatty acid metabolism, degradation of valine, leucine and isoleucine, as well as tryptophan metabolism. However, these associations were based on only one-fifth of the genes, with the majority of downregulated genes not yet assigned to functions or pathways.

### Cytokine and pattern recognition receptor (PRR) gene expression during infection

**Fig. 4:**
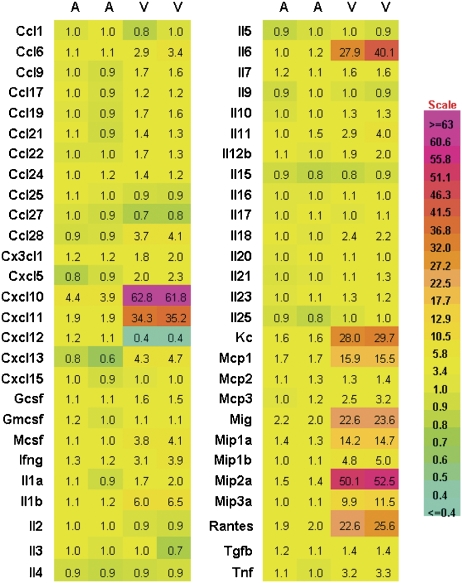
Cytokine/chemokine gene expression in infected kidneys. For each cytokine, the gene expression levels obtained by microarray analyses are shown relative to control, where A represents infection by the attenuated strain and V represents infection by the virulent strain. Expression level (-fold) change is colored as shown on the scale.

Because of the differences in renal gene expression associated with cytokine interactions and signaling in the different infections, a more detailed examination of cytokine gene expression was performed (Fig. 4). Results demonstrated a clear induction of the proinflammatory cytokine genes Il1a and b, Il6 and Tnf, which have also been linked to the acute-phase response, in the kidneys of mice infected with the virulent strain, whereas little induction of these genes was seen in kidneys infected with the attenuated strain. Among 54 cytokine genes examined, 32 were significantly altered in virulent strain infection compared with control kidneys (Fig. 4). The majority of these genes were upregulated, but a single cytokine gene, Cxcl12, was downregulated in the renal response to the virulent *C. albicans* strain and unchanged in attenuated strain infection.

In the kidneys of mice infected with the attenuated strain, the only cytokine/chemokine genes upregulated were Cxcl10, Cxcl11, Kc, Mcp1, Mig, Mip1a, Mip2a and Rantes. These genes, along with Il6, were also the most upregulated in virulent strain infection, with expression levels at least 10-fold higher than those of mice infected with the attenuated strain (Fig. 4).

The large differences in cytokine gene expression in response to *C. albicans* infection prompted an examination of the expression of PRR genes, the signaling from which influences cytokine production. PRRs known to be involved in the recognition of *C. albicans* include Toll-like receptors (TLRs) (Netea *et al.*, 2006a,b; Carpenter & O'Neill, 2007;) and C-type lectins (Willment & Brown, 2008).

**Fig. 5:**
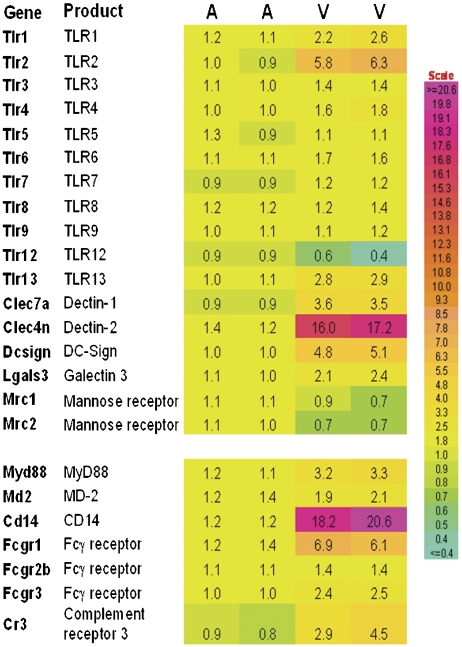
PRR gene expression in infected kidneys. The expression levels of various pattern recognition receptors and some adaptor molecules are shown, where A represents infection by the attenuated strain and V represents infection by the virulent strain. Expression levels are normalized to controls. Expression level (-fold) change is colored as shown on the scale.

There was little change in PRR gene expression in kidneys infected with the attenuated strain (Fig. 5), but in the kidneys of mice infected with the virulent strain there was an increased expression of genes encoding TLRs (Tlr1, Tlr2, Tlr4, Tlr6 and Tlr13) and their associated adaptor molecules: Myd88, Cd14 and Md2 (Fig. 5). The greatest increase in TLR gene expression was seen for Tlr2. In addition, a mouse-specific TLR gene, Tlr12, was downregulated during virulent strain infection, but was unaltered in attenuated strain infection.

Although the majority of non-TLR PRR genes examined showed increased expression in the kidneys of virulent strain-infected mice, the expression of mannose receptor genes was reduced, and the greatest increase in non-TLR PRR gene expression was seen for dectin-2 (Fig. 5). Similarly, expression of FCγ receptor genes, whose products are required for dectin-2 signaling (Sato *et al.*, 2006), were also upregulated in response to virulent strain infection.

### Confirmation of transcript profiling results by qRT-PCR

**Fig. 6:**
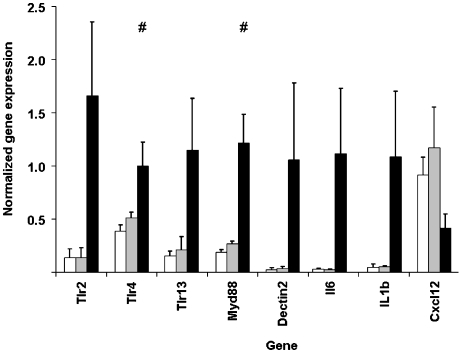
qRT-PCR confirms microarray gene expression differences. qRT-PCR was used to analyze gene expression of immune-related genes in individual kidney samples. mRNA copy number for each gene was normalized to a GAPDH control. Data represent the mean of three individual samples (± SD) for each treatment group. White bars represent control kidneys; gray bars, attenuated strain-infected kidneys and black bars, virulent strain-infected kidneys. Means were compared by Kruskal–Wallis and Mann–Whitney-*U*-tests. For all genes, *P*<0.001 for control vs. virulent and attenuated vs. virulent. For control vs. attenuated, ^#^*P*<0.05 for control vs. attenuated.

In order to confirm the gene expression differences found by microarray analysis, qRT-PCR was used to analyze expression levels of several genes of interest. Immune response-associated genes, with altered expression in virulent strain infection, were selected for further investigation: Tlr2, Tlr4, Tlr13, Myd88, Il1b, Il6, dectin-2 and Cxcl12 (Fig. 6).

Results obtained by qRT-PCR for the individual kidney RNA samples showed good correlation with the transcript profiling results for RNA pools (*r*=0.93, data not shown), with statistically significant differences found for all genes when renal responses to the virulent strain were compared with those of mice infected with the attenuated strain or of control mice (Fig. 6). These results confirmed the changes in innate immune response-related gene expression seen by microarray analysis in kidneys in response to the virulent strain, but that only occurred to a limited extent in response to the attenuated strain.

Surprisingly, qRT-PCR also demonstrated a small, but significant, increase in Tlr4 gene expression in response to infection by the attenuated strain that was not seen by microarray analysis (Fig. 6).

## Discussion

The mouse intravenous challenge model is frequently used to characterize the virulence potential of *C. albicans* isolates and strains and is a good model of severe human disseminated infection. From previous studies, we have shown that kidney burdens in virulent strain-infected mice continue to increase until the mice become severely ill (MacCallum & Odds, 2005). In contrast, in infection initiated by the attenuated strain, kidney burdens remain roughly constant over 28 days of infection (Bates *et al.*, 2005; MacCallum *et al.*, 2009;). These results demonstrate that the virulent strain initiates a progressive infection, while infection initiated by the attenuated strain is controlled. Analysis of organ burdens at 24 h postinfection demonstrated virulence differences between the two strains, even at this early time in infection. Kidney burdens were significantly higher for mice infected with the virulent strain and infiltrating immune cells were already evident in the kidneys of these mice. These results clearly indicate that early renal responses to the two *C. albicans* strains must differ.

In this study, the transcriptional responses of the kidney at 24 h postinfection were analyzed to determine how responses to known virulent and attenuated *C. albicans* strains differ. Microarray gene expression patterns were confirmed by qRT-PCR, confirming that the use of RNA pools for transcript profiling is a valid approach for this experimental infection model.

Results clearly demonstrated that the kidney responds to *C. albicans* infection by inducing a core response: the acute-phase response and complement and coagulation cascades. Induction of the acute-phase response was not surprising as this reaction is a response to physiological alterations and it is usually activated during inflammation. Although previously associated with the liver (Kushner, 1982), renal proximal tubular epithelial cells have also been shown to generate a strong acute-phase response (Luyckx *et al.*, 2009), suggesting that this cell type may be involved in the core response.

While the core response appeared sufficient to prevent overgrowth of the attenuated strain within the first 24 h of infection, reducing fungal burdens to levels similar to those found at 28 days postinfection (Bates *et al.*, 2005), it was insufficient to control the virulent strain. Deficiencies in the core response to *C. albicans* infection have previously been shown to lead to increased susceptibility to systemic candidiasis (Gelfand *et al.*, 1978; Odds, 1988; Mullick *et al.*, 2004b; Held *et al.*, 2008;), and may allow normally attenuated strains to initiate progressive infections.

In progressive renal infection initiated by the virulent strain, the fungus proliferated in the kidneys, accompanied by a massive transcriptional change, mostly genes associated with innate and adaptive immune responses. This demonstrates a clear link between induction of host immune responses in the kidney and progression of *C. albicans* infection. Immune responses occurring in the kidney have previously been linked to *C. albicans* infection outcome (Brieland *et al.*, 2001; Spellberg *et al.*, 2003; Cao *et al.*, 2005; MacCallum *et al.*, 2009;).

The majority of cytokines and chemokines genes examined showed increased expression in response to infection by the virulent strain, their products thus leading to attraction and activation of immune cells. In contrast, the attenuated strain showed little induction of proinflammatory gene expression in the kidney, correlating with the lack of infiltrating immune cells in the kidney (Fig. 1b) and also a previous study where the attenuated strain induced much lower levels of tumor necrosis factor (TNF)-α and IL-6, due to its defect in cell wall protein mannosylation (Netea *et al.*, 2006b). However, although the defect in TNF-α and IL-6 induction is due to the lack of mannosylation in the attenuated *pmr1*Δ mutant1 (Netea *et al.*, 2004), we have recently demonstrated that attenuated clinical isolates, in general, induce lower renal levels of chemokines and proinflammatory cytokines compared with virulent isolates (MacCallum *et al.*, 2009). This confirms that upregulation of cytokines and chemokines is a general response of kidneys exposed to virulent *C. albicans* strains (MacCallum *et al.*, 2009).

Proinflammatory cytokines, for example TNF-α and interferon (IFN)-γ, are required for normal responses to *C. albicans* infection (Steinshamn & Waage, 1992; Kullberg *et al.*, 1993; Louie *et al.*, 1994; Brieland *et al.*, 2001;). However, previous studies have demonstrated that the fatal outcome of *C. albicans* infection is linked to the development of T-helper type 2 (Th2) and IL-10 responses in the kidney (Brieland *et al.*, 2001; Spellberg *et al.*, 2003; Cao *et al.*, 2005;). IL-10 is known to have strong inhibitory effects on defense against *C. albicans* (Romani *et al.*, 1994), with IL-10 knockout mice more resistant to *C. albicans* infection (Vazquez-Torres *et al.*, 1999). We found, in agreement with others (Brieland *et al.*, 2001; Spellberg *et al.*, 2003;), that renal expression of Il10 was induced in progressive infection. Cytokine profiles, however, showed evidence of both Th1 and Th2 responses in infected kidneys, similar to the results obtained by Bellocchio *et al.* (2004). Differences between this study and the study of Cao *et al.* (2005), who clearly found that a Th2 response in the kidney was linked to a lethal outcome, may be due to the use of different *C. albicans* strains, or due to different kinetics of infection progression, as mice in this study had lower survival times than those in the study of Cao *et al.* (2005).

The renal transcriptional response to *C. albicans* progressive infection also included regulation of PRR gene expression. Previously, a number of PRRs have been demonstrated to be involved in host recognition of *C. albicans* (Netea *et al.*, 2006a; Willment & Brown, 2008;), particularly TLR2, TLR4 (Netea *et al.*, 2002, 2008; Villamon *et al.*, 2004b; Blasi *et al.*, 2005) and dectin-1 (Brown *et al.*, 2003; Gantner *et al.*, 2005; Gow *et al.*, 2007; Ferwerda *et al.*, 2008;). In this study, the kidney cells upregulating expression of the PRRs were not identified but, in response to renal injury, TLR2 and TLR4 levels are increased on tubular epithelial cells (Wolfs *et al.*, 2002; Kim *et al.*, 2005a; Leemans *et al.*, 2005; Shigeoka *et al.*, 2007; Wu *et al.*, 2007;). This again suggests that these cells may play a role in renal responses to *C. albicans* infection, especially as these cells are capable of producing numerous cytokines and chemokines (reviewed in Daha & van Kooten 2000), and have been shown to respond to other infections with induction of inflammatory cascades (Ross *et al.*, 2006). However, the microarray results in this study also show good agreement with those found for endothelial cells (Muller *et al.*, 2007), indicating that transcriptional changes may be due to multiple cell types in the kidney. Examination of the transcriptional responses of kidney cell subsets to *C. albicans* infection will be the subject of future studies.

In response to infection by the attenuated strain, there was little change in PRR gene expression in the kidneys by microarray analysis. However, qRT-PCR indicated a small significant increase in Tlr4 expression in these kidneys. This demonstrated that, as shown previously (Allanach *et al.*, 2008), qRT-PCR is more sensitive in detection of subtle changes in gene expression and is more suitable for investigating small gene expression differences.

The PRR genes most highly upregulated in response to infection by the virulent strain were Tlr2 and a C-type lectin gene, dectin-2, which is involved in the recognition of *C. albicans* hyphae (McGreal *et al.*, 2006; Sato *et al.*, 2006;). TLR2 has also been implicated in the recognition of *C. albicans* hyphae (van der Graaf *et al.*, 2005) and plays an important role in *C. albicans* infection (Netea *et al.*, 2002; Blasi *et al.*, 2005; Gil & Gozalbo, 2006; Murciano *et al.*, 2007;). Many of the cytokine/chemokine genes involved in the response to virulent strain infection have been shown to require signaling from TLR2 in combination with dectin-1 (Brown *et al.*, 2003; van der Graaf *et al.*, 2005; Netea *et al.*, 2006b; Ferwerda *et al.*, 2008;). However, in this study, although dectin-1 was upregulated in response to infection by the virulent strain, dectin-2 was much more highly upregulated. Differences in the expression of C-type lectins between the different studies are most likely due to the host cells analyzed, with whole kidneys analyzed in this study and isolated immune cells analyzed in others.

One of the major questions arising from this study is why infection by the virulent strain in the kidney is not controlled when there is an obvious inflammatory response occurring. This may be due to induction of regulatory responses in the kidneys. Although TLR2 signaling can induce the production of proinflammatory mediators (macrophage inflammatory protein-2, KC (keratinocyte-derived cytokine), TNF-α, IL-1β and IFN-γ) (Netea *et al.*, 2002, 2004; Bellocchio *et al.*, 2004; Villamon *et al.*, 2004a, b; van der Graaf *et al.*, 2005; Gil & Gozalbo, 2006; Murciano *et al.*, 2007; De Filippo *et al.*, 2008), it can also induce IL-10 production, suppressing immune responses to *C. albicans* (Romani *et al.*, 1994, 2004; Netea *et al.*, 2004). TLR2 is also involved in controlling the expansion and function of regulatory T cells (Netea *et al.*, 2004; Sutmuller *et al.*, 2006;), with fewer regulatory T cells found in TLR2 knockout mice (Netea *et al.*, 2004). Therefore, increased expression of Tlr2, leading to increased levels of IL-10 and increased survival of regulatory T cells, may dampen the immune response against *C. albicans*, even in the presence of immune infiltrates in the kidney, and allow infection to progress.

Amplification of immune responses to infection in the kidney may also occur, as the proinflammatory cytokines TNF-α and IFN-γ have been shown to induce renal expression of Tlr2 and Tlr4 (Wolfs *et al.*, 2002). This may lead to continued production of both proinflammatory and immunosuppressive mediators in response to fungal cells. Continued inflammatory responses, where the fungal growth is not controlled, can be regarded as inappropriate, and have previously been shown to contribute to development of sepsis (reviewed in Sriskandan & Altmann, 2008), which leads to host deterioration and eventually death in the mouse model of systemic *C. albicans* infection (Spellberg *et al.*, 2005).

This study has several limitations, including study of a single time point during infection and the use of single examples of virulent and attenuated *C. albicans* strains; however, it does serve as a basis for future expansion in this area of research, examining the renal responses temporally during infection progression, in different mouse strains (including mouse strains with defined gene knockouts) and responses in organs other than the kidney, where infection by *C. albicans* is known to be controlled.

## Conclusion

This study has characterized the transcriptional profiles of kidneys responding to infection by virulent and attenuated *C. albicans* strains. A core response was identified, which consisted of the acute-phase response, including the complement and coagulation cascades. Progressive infection initiated by the virulent *C. albicans* strain was characterized by a strong inflammatory transcriptional response, with massive induction of cytokine and chemokine gene expression, accompanied by an influx of immune cells into the infected kidney. The induced renal immune response did not control growth of the virulent *C. albicans* strain *in vivo*, but possibly contributes to the development of sepsis, causing host death. In the future, therapies targeting the immune response to progressive infection may be effective in treating systemic *C. albicans* infection.

## Statement

Re-use of this article is permitted in accordance with the Terms and Conditions set out at: http://www3.interscience.wiley.com/authorresources/onlineopen.html
